# Perceived Stress Among Resident Doctors in Jordanian Teaching Hospitals: Cross-Sectional Study

**DOI:** 10.2196/14238

**Published:** 2019-10-02

**Authors:** Nizar Maswadi, Yousef S Khader, Ahmad Abu Slaih

**Affiliations:** 1 Department of Parasitic and Zoonotic Diseases Directorate of Communicable Diseases Ministry of Health Amman Jordan; 2 Department of Community Medicine, Public Health and Family Medicine Jordan University of Science & Technology Irbid Jordan; 3 Community Medicine Residency Program Jordan Ministry of Health Amman Jordan

**Keywords:** psychology, physicians, teaching hospitals, Jordan

## Abstract

**Background:**

Medical residents in Jordanian hospitals are involved in many clinical and nonclinical tasks that expose them to various stress factors. High stress and burnout have the potential to negatively impact work performance and patient care, including medication errors, suboptimal care, clinical errors, and patient dissatisfaction.

**Objective:**

This study aimed to determine the perceived stress among medical residents in Jordanian hospitals and its associated risk factors.

**Methods:**

A cross-sectional study was conducted among residents in Jordanian hospitals. A cluster sample of 5 hospitals with residency programs was selected from different health sectors. All residents who were working in the selected hospitals were invited to participate in this study, during the period from April to July 2017. A total of 555 residents agreed to participate in this study, giving a response rate of 84%. The perceived stress scale (PSS) was used for assessment.

**Results:**

A total of 398 male and 157 female residents were included in this study. The mean PSS score in this study was 21.6; 73% (405/555) of the residents had moderate level of stress, and 18% (100/555) had high level of stress. About 6.7% (37/555) of the residents had hypertension, 2.7% (15/555) had diabetes, 3.2% (18/555) had heart disease, and 8.5% (47/555) were anemic. 233 (42%) respondents complained of back pain, and 161 (29%) of the respondents complained of insomnia. Stress was associated with higher workload, sleep deprivation, and dissatisfaction in the relationship with colleagues, with income, and with the program. In multivariate analysis, the following factors were significantly associated with stress: female gender, dissatisfaction with working environment, and facing work-related, academic, and family stressors.

**Conclusions:**

The majority of medical residents in Jordanian hospitals felt nervous and stressed. Conducting stress management programs during residency and improving the work environment are strongly recommended.

## Introduction

Stress and psychosocial risk factors are considered critical issues in the field of occupational health [1]. Occupational stress is understood as the experience of stress that is caused by factors within the occupation or job. Occupational stress is a rational, physical, and emotional deterioration that is brought about by the dissimilarities between the job requirements and the personal skills, capabilities, and competencies. The impact of stress on the physical and mental health, as well as the productivity of both the organizations and the employees, is a growing concern of organizations. High stress and burnout have the potential to negatively impact work performance and patient care, including medication errors, suboptimal care, clinical errors, and patient dissatisfaction [2,3]. Many studies have looked into the stress and the burnout levels of medical personnel, and an extremely high level of stress has been observed in hospital nurses, surgeons, general practitioners, and resident doctors [4,5]. Physicians are exposed to many stressors, such as the burden imposed by the expectations of a high degree of professionalism, responsibility for patient well-being, and maintenance of relationships with patients and health workers, as well as concerns about medical errors and malpractice litigation [6]. Studies have demonstrated that up to 76% of residents meet the criteria for burnout and that they have expressed career dissatisfaction, as well as concern that they provide suboptimal patient care [1]. Residents report that the working conditions they are subjected to during residency lead to reduced attention, empathy, concern, and sensitivity, and increased irritability, abruptness, and a tendency to objectify patients [7]. The significance of this study emerges from the fact that the quality of health care can be extremely influenced by the stressed health staff. Residency involves long hours, large numbers of patients, and sleep deprivation [8,9]. When the level of stress exceeds a critical level, it can manifest as distress, resulting in psychological morbidity, impairment, and burnout [10]. This study aimed to assess the level of stress among residents and determine the main leading factors causing stress among resident doctors in Jordanian hospitals.

## Methods

### Study Design

This cross-sectional study was conducted among medical residents trained in different residency programs in Jordan, during the period from April to July 2017. All residents who were enrolled in different sectors, including public, private, and teaching programs, were eligible to be included in the study. Al-Bashir and Prince Hamza hospitals were selected from the public sector, Islamic hospital and Jordan hospital were selected from the private sector, and King Abdullah University hospital was selected from the teaching health sector. All residents who were working in the selected hospitals were invited to participate in this study. A total of 555 residents agreed to participate in this study, giving a response rate of 84%. The ethical approval was obtained from the Institutional Review Board at the Ministry of Health in Jordan.

### Data Collection

A self-administrated questionnaire was used to collect the data. The questionnaire included data on sociodemographic characteristics (age, gender, and marital status), residency characteristics (specialty and year), diseases or symptoms experienced by medical residents in the last year, workload (number of inpatients and outpatients treated per day, sleep duration, and quality), recently faced stressors (work related and nonwork related), and job satisfaction. Perceived stress scale (PSS) was used to assess residents’ perception of stress over the past month [11]. The respondents answered each PSS question on a Likert-type scale (never, almost never, sometimes, fairly often, or very often). We scored the answers to questions 1, 2, 3, 6, 9, and 10 by giving a score of 0 to the “never” answers and 4 to the “very often” responses. Questions 4, 5, 7, and 8 were scored by scoring “never” as 4 and “very often” as 0. The PSS score was calculated by summing up the scores of all the individual questions. Scores ranging from 0 to 13 would be considered low stress, scores ranging from 14 to 26 would be considered moderate stress, and scores ranging from 27 to 40 would be considered high perceived stress, with higher scores indicating higher levels of stress. The PSS had good internal consistency among its items, as indicated by an overall Cornbrash alpha value of .76. The study questionnaire was pilot tested on 30 participants (n=30). Necessary changes on the wording and phrasing of the questions were revised according to the pilot study findings. The face and content validity of the study questionnaire were evaluated by 3 experts.

### Statistical Analysis

Data were coded and entered through IBM SPSS, version 20, software. Data were described using percentages and means. Variables were displayed through percentage frequency tables. Comparisons between 2 means and more than 2 means were tested for statistical significance by using an independent *t* test and a 1-way analysis of variance, respectively. General linear model was used to test the factors associated with stress level in the multivariate analysis. A *P* value <.05 was considered statistically significant.

## Results

### Characteristics of the Participants

A total of 398 (71.7%, 398/555) male and 157 (28.3%, 157/555) female residents were included in this study. Table 1 shows their sociodemographic and work characteristics.

The average (SD) age was 30.0 (3.0) years. More than half of respondents were residents in the fields of surgery, internal medicine, pediatrics, and radiology. A majority of the participants (92%, 511/555) worked night shift, and 95.3% (529/555) dealt with emergency cases. The median number of inpatients and outpatients treated by the residents per day was 15 and 35 patients, respectively. Approximately 78.9% (435/555) of the residents reported that they slept for less than 6 hours per day, and only 21.4% (119/555) of the residents reported feeling refreshed after sleep.

Depending on self-reported data, about 6.7% (37/555) of the residents had hypertension, 2.7% (15/555) had diabetes, 3.2% (18/555) had heart disease, 8.5% (47/555) were anemic, and 7.0% (39/555) had lung or breathing problems. 233 (42%) respondents complained of back pain, 161 (29%) complained of insomnia, 83 (15%) of stomach ulcer, 78 (14%) of gastritis, 67 (12%) of emotional problems, and 50 (9%) colitis.

**Table 1:** The sociodemographic and work characteristics of medical residents in Jordanian hospitals (N=555).

Characteristics		Statistics, n (%)
**Gender**		
	Male	398 (71.7)
	Female	157 (28.3)
**Age (years)**		
	<30	267 (51.0)
	≥30	257 (49.0)
**Marital status**		
	Married	323 (58.2)
	Single	232 (41.8)
**Specialty**		
	Surgery	110 (19.8)
	Internal medicine	85 (15.3)
	Pediatrics	84 (15.1)
	Radiology	61 (11.0)
	Anesthesia	43 (7.7)
	Obstetrics and gynecology	38 (6.8)
	Orthopedics	35 (6.3)
	Others	99 (17.8)
**Residency year**		
	First	162 (29.2)
	Second	124 (22.3)
	Third	104 (18.7)
	Fourth	103 (18.6)
	Fifth	62 (11.2)
**Night shift per month**		
	1-5	133 (24.0)
	>5	378 (68.1)
Deal with emergency cases		529 (95.3)
**Inpatients treated per day**		
	≤15	282 (50.1)
	>15	241 (49.9)
**Outpatients treated per day**		
	≤35	258 (50.1)
	>35	257 (49.9)
**Sleep duration (hours)**		
	≤5	225 (40.8)
	6	210 (38.1)
	7	80 (14.5)
	≥8	36 (6.5)
Feeling refreshed after sleep		119 (21.4)

### Stressors and Satisfaction

Table 2 shows stressors, job satisfaction, and ideation among the study participants. The most commonly reported stressors included work-related, financial, academic, and family stressors.

About 70.0% (388/555) of the participants were satisfied with their relationship with colleagues; 21.1% (117/555) of the participants were satisfied with the training program, whereas 14% (78/555) of the participants were dissatisfied. A total of 15.7% (87/555) of the residents frequently considered changing their specialty, and 23.6% (131/555) had frequent thoughts of quitting the medical profession.

**Table 2:** Stressors, job satisfaction, and ideation experienced by medical residents in Jordanian hospitals.

Characteristics		Statistics, n (%)
Comfortable working environment		86 (15.5)
Satisfied with income		30 (5.4)
**Facing stressors experienced in the last month**		
	Work related	479 (86.3)
	Financial	345 (62.2)
	Academic	290 (52.3)
	Family	126 (22.7)
	Alienation	74 (13.3)
	Death	19 (3.4)
	Others	82 (14.8)
Satisfied with the relationships with colleagues		388 (70.0)
Satisfied with training program		117 (21.1)
**Thoughts of changing specialty**		
	Very often	87 (15.7)
	Sometimes	198 (35.7)
	Rarely	104 (18.7)
	Never	166 (29.9)
**Thoughts of quitting**		
	Very often	131 (23.6)
	Sometimes	205 (36.9)
	Rarely	94 (16.9)
	Never	125 (22.5)

### Responses to Perceived Stress Scale

A majority of the medical residents (73%, 405/555) had moderate level of stress, 18% (100/555) of the medical residents had high level of stress, and 9% (50/555) had mild level of stress. Table 3 shows the responses to PSS.

During the last month preceding the survey, 76.2% (423/555) of the residents often (“fairly” or “very”) felt nervous and stressed, 44.8% (249/555) felt upset because of unexpected events, 45.8% (254/555) felt angered by circumstances that were beyond their control, 42.4% (235/555) felt unable to control important things in their life, and 44.1% (245/555) felt that difficulties were piling up, which were too high to overcome. In contrast, 34.7% (193/555) of the residents often felt that things were going their way, 20.5% (114/555) felt that they were on top of things, 19.7% (109/555) had often been able to control irritations in their life, and 15.4% (85/555) often felt confident in their ability to handle personal problems.

**Table 3:** Medical residents’ responses to perceived stress scale in Jordanian hospitals.

Perceived stress scale items	Never, n (%)	Almost never, n (%)	Sometimes, n (%)	Fairly often, n (%)	Very often, n (%)
In the last month, how often have you been upset because of something that happened unexpectedly?	41 (7.4)	63 (11.4)	202 (36.4)	150 (27.0)	99 (17.8)
In the last month, how often have you felt that you were unable to control the important things in your life?	44 (7.9)	96 (17.3)	180 (32.4)	147 (26.5)	88 (15.9)
In the last month, how often have you felt nervous and “stressed?”	8 (1.4)	19 (3.4)	105 (18.9)	197 (35.5)	226 (40.7)
In the last month, how often have you felt confident about your ability to handle your personal problems?	99 (17.8)	178 (32.1)	193 (34.8)	68 (12.3)	17 (3.1)
In the last month, how often have you felt that things were going your way?	25 (4.5)	104 (18.7)	233 (42.0)	129 (23.2)	64 (11.5)
In the last month, how often have you found that you could not cope with all the things that you had to do?	31 (5.6)	107 (19.3)	234 (42.2)	135 (24.3)	48 (8.6)
In the last month, how often have you been able to control irritations in your life?	66 (11.9)	195 (35.1)	185 (33.3)	82 (14.8)	27 (4.9)
In the last month, how often have you felt that you were on top of things?	53 (9.5)	179 (32.3)	209 (37.7)	79 (14.2)	35 (6.3)
In the last month, how often have you been angered because of things that were outside of your control?	22 (4.0)	75 (13.5)	204 (36.8)	171 (30.8)	83 (15.0)
In the last month, how often have you felt difficulties were piling up so high that you could not overcome them?	19 (3.4)	96 (17.3)	195 (35.1)	164 (29.5)	81 (14.6)

### Thoughts of Changing Specialty and Quitting Work According to Stress Level

**Figure 1:**
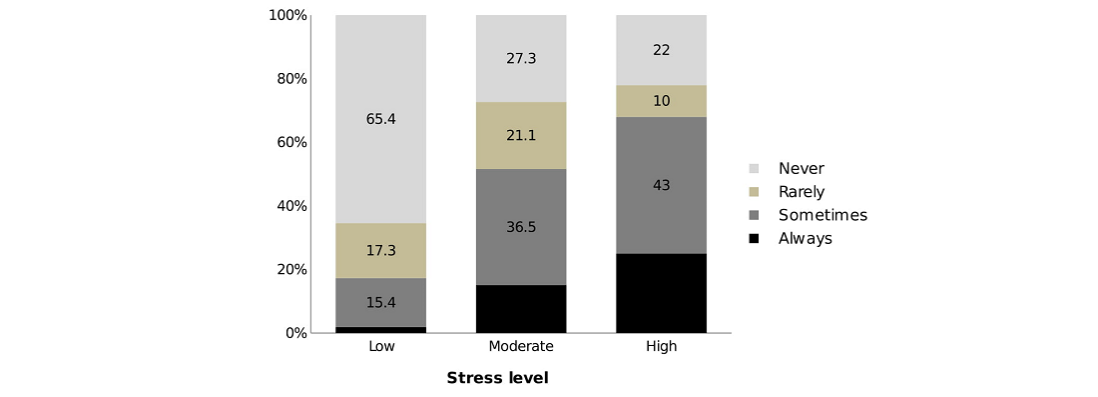
Percentage of residents who thought of changing specialty according to stress level.

**Figure 2:**
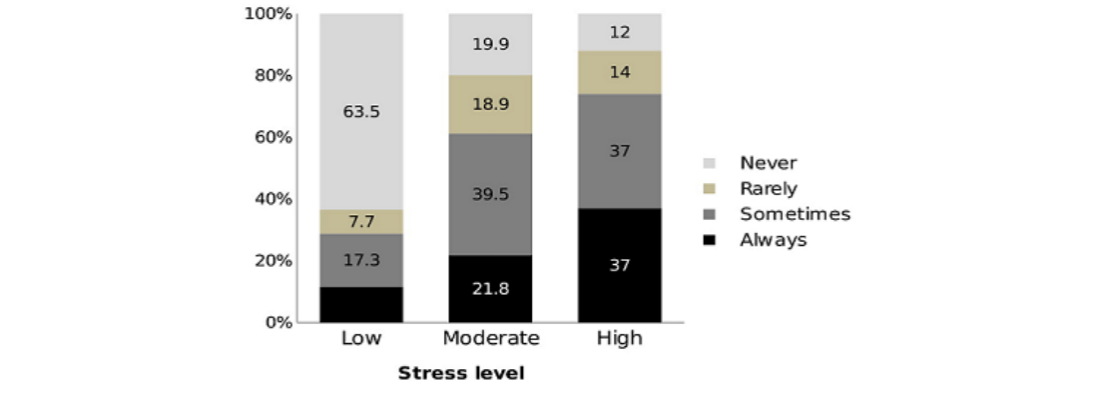
Percentage of residents who thought quitting work according to stress level.

Figure 1 shows that a quarter of the residents with high level of stress were always thinking of changing specialty, whereas 43% (239/555) of the residents were sometimes thinking of changing specialty. About two-thirds of the residents (361/555, 65%) with low level of stress had never thought of changing specialty. Figure 2 shows that 37% (205/555) of the residents with high level of stress were always thinking of quitting work, whereas two-thirds (350/555, 63%) of the residents with low level of stress had never thought of quitting work.

### Stress Level and Its Associated Factors

The mean (SD) of the PSS scores was 21.6 (5.8). The average PSS scores, according to sociodemographic and work characteristics and level of satisfaction, are shown in Table 4.

The average PSS score differed significantly according to gender, specialty, night shifts, number of inpatients treated per day, number of outpatients treated per day, satisfaction with working environment, satisfaction with income, satisfaction with the relationships with colleagues, and satisfaction with training program. Table 5 shows the average PSS score according to stressors experienced by residents. Stress level was significantly associated with work-related stressors, financial stressors, academic stressors, and family-related stressors.

Table 6 shows multivariate analysis of factors associated with mean PSS score. Stress level was significantly higher among females, in those working in uncomfortable environments, and in those with work-related, academic-related, and family-related stressors. Those with anemia also had a higher stress level.

**Table 4:** The average perceived stress scale score according to sociodemographic and work characteristics and level of satisfaction.

Characteristics		Mean (SD)	*P* value
**Age (years)**			**.49**
	<30	21.8 (5.68)	—^a^
	≥30	21.4 (5.86)	—
**Gender**			**<.001**
	Male	21.0 (5.69)	—
	Female	23.3 (5.88)	—
**Marital status**			**.74**
	Married	21.7 (5.63)	—
	Single	21.5 (6.13)	—
**Specialty**			**.02**
	Surgery	21.6 (6.30)	—
	Internal medicine	22.2 (5.98)	—
	Pediatrics	22.2 (5.55)	—
	Radiology	20.5 (6.42)	—
	Anesthesia	19.2 (4.92)	—
	Obstetrics and gynecology	23.6 (5.43)	—
	Orthopedics	22.6 (5.14)	—
	Others	21.4 (5.49)	—
**Residency years**			**.33**
	First	21.3 (5.68)	—
	Second	22.5 (5.93)	—
	Third	21.8 (5.96)	—
	Fourth	21.2 (5.94)	—
	Fifth	21.0 (5.64)	—
**Emergency cases**			**.65**
	Yes	21.6 (5.79)	—
	No	22.1 (6.74)	—
**Night shift**			**.005**
	1-5	20.2 (6.17)	—
	>5	22.0 (5.69)	—
**Inpatients treated per day**			**.02**
	≤15	21.0 (6.02)	—
	>15	22.3 (5.61)	—
**Outpatients treated per day**			**.007**
	≤35	20.8 (5.88)	—
	>35	22.2 (5.56)	—
**Comfortable working environment**			**<.001**
	Yes	17.7 (6.16)	—
	No	22.3 (5.49)	—
**Satisfied with income**			**.002**
	Yes	18.4 (5.94)	—
	No	21.8 (5.78)	—
**Satisfied with the relationships with colleagues**			**<.001**
	Satisfied	20.9 (5.89)	—
	Dissatisfied	23.5 (5.30)	—
	Not sure	23.4 (5.36)	—
**Satisfied with training program**		**—**	**<.001**
	Satisfied	19.2 (5.91)	—
	Dissatisfied	22.6 (5.67)	—
	Not sure	21.4 (5.32)	—

^a^Not applicable.

**Table 5:** The average perceived stress scale score according to stressors experienced by medical residents in Jordanian hospitals.

Characteristics		Mean (SD)	*P* value
**Work related**			**<.001**
	Yes	22.2 (5.53)	—^a^
	No	18.1 (6.47)	—
**Financial**			**<.001**
	Yes	22.5 (5.92)	—
	No	20.2 (5.42)	—
**Academic**			**<.001**
	Yes	23.0 (5.65)	—
	No	20.1 (5.69)	—
**Family**			**<.001**
	Yes	23.8 (5.41)	—
	No	21.0 (5.81)	—
**Alienation**			**.65**
	Yes	21.9 (5.92)	—
	No	21.6 (5.83)	—
**Death**			**.12**
	Yes	23.7 (6.25)	—
	No	21.6 (5.81)	—

^a^Not applicable.

**Table 6:** Multivariate analysis of factors associated with mean perceived stress scale score.

Characteristics		Mean (SE)	95% CI	*P* value
**Gender**				**.002**
	Male	20.2 (0.6)	19.0-21.3	—^a^
	Female	22.0 (0.6)	20.9-23.2	—
**Specialty**				**.15**
	Anesthesia	19.3 (0.9)	17.5-21.1	—
	Radiology	21.1 (0.8)	19.6-22.7	—
	Surgery	21.7 (0.7)	20.3-23.1	—
	Internal medicine	22.0 (0.8)	20.5-23.5	—
	Obstetric	20.8 (1.0)	18.8-22.7	—
	Orthopedic	22.2 (1.0)	20.2-24.3	—
	Pediatric	20.7 (0.7)	19.3-22.2	—
	Others	21.0 (0.7)	19.7-22.3	—
**Comfortable working environment**				**<.001**
	Yes	19.2 (0.7)	17.8-20.5	—
	No	23.1 (0.5)	22.0-24.1	—
**Work related**				**<.001**
	Yes	22.4 (0.5)	21.4-23.8	—
	No	19.9 (0.7)	18.5-21.3	—
**Academic related**				**<.001**
	Yes	22.2 (0.5)	21.1-23.2	—
	No	20.0 (0.5)	18.9-21.2	—
**Family related**				**.001**
	Yes	22.0 (0.6)	20.7-23.3	—
	No	20.2 (0.5)	19.2-21.2	—
**Anemia**				**.007**
	Yes	22.3 (0.8)	20.6-23.9	—
	No	19.9 (0.4)	19.1-20.8	—

^a^Not applicable.

## Discussion

Work-related stress is very common among health care workers. Health care providers around the world are subject to pressures resulting from a sharp escalation of change, growing economic pressures, technological advances, increasing patient expectations, rationing of health care, and the requirement for more evidence-based and high-quality health care, improved performance, and productivity. It is well documented that health workers experience higher levels of stress and stress-related health problems than other occupational groups [12].

To the best of our knowledge, this study was the first to determine the magnitude of perceived stress among medical residents in Jordan. The study included residents of various specialties from different health sectors.

The study showed that the majority of medical residents had a moderate level of stress (405/555, 73%), and 18% (100/555) of the medical residents had a high level of stress. The perceived stress among residents in this study was comparable with the perceived stress reported among residents in other parts of the world. The mean PSS score in this study was 21.6. This mean is almost the same score for 938 medical residents in Saudi Arabia, where they reported a mean score of 22 [13]. Similarly, the mean PSS score was 21.7 in 106 cardiology residents in Argentina [14], and 19.9 among 159 anesthesia residents in Turkey [15]. A lower score of 16.1 had been reported among 168 family medicine residents in the United States [16]. On the other hand, a study among 84 doctors working in a tertiary care teaching hospital in India reported a mean of 18.3, and another study reported a mean of 18 among 303 physicians working in an Asser region in Saudi Arabia [17-19].

The variations in the PSS score in different studies might be explained by many factors, such as working environment, the specialty, and differences in sociodemographic and cultural characteristics. However, the variations among these studies are not clinically significant.

During the 30 days preceding the survey, 76.2% (423/555) of the residents (“fairly” or “very”) felt nervous and stressed, which can be considered higher than other countries in the region, such as Saudi Arabia, where the 68.2% of the medical residents reported being under stress. On the other side, 19.7% (109/555) of the residents in our sample had often been able to control irritations in their life, in comparison with 34.7% of the Saudi residents [13]. Unfortunately, there is a lack of studies that examine stress among residents in Jordan; therefore, the study compares the findings with the perceived stress among nursing students in Jordan, as the mean of the PSS was 45.9 [20].

According to stress and the personal characteristics, female residents had a significantly higher level of stress than male residents, which was identified by multiregression analysis. There are several studies in agreement with our findings [21,22]. The reasons for this difference should be considered, as more serious consequences might occur among the female physicians who face more workplace adversity compared with males experiencing the same level of occupational stress, in terms of mistrust from the patients, as well as having the dual responsibilities of career and family.

In our multivariate results, stress was significantly associated with anemia; one of the most common symptoms of anemia is a feeling of fatigue and a lack of energy, and although the pathogenesis of anemia-related fatigue remains unclear, some suggest that abnormalities in energy metabolism play a role in inducing fatigue [23]. The relationship between anemia and fatigue is universally accepted. However, early studies were unable to show a clear association between fatigue and hemoglobin levels. There was no evidence of an association between iron deficiency and fatigue in the absence of anemia, suggesting that iron deficiency is not a clinically relevant contributor to fatigue. This gives an important insight for the need to conduct further investigations on the association between anemia and fatigue.

Stress was significantly associated with obstetrics and gynecology residents, surgery, internal medicine, and pediatrics in most countries. It is a well-known fact that the obstetrics and gynecology residency has the highest prevalence of burnout among all specialties [22]. This is explained by the fact that residency in obstetrics and gynecology in most countries is characterized by sleep deprivation, long weekly working hours, postcall clinical responsibilities, and professional liability insurance crises.

The residents in this study, who shouldered higher workloads (dealing with more patients and working more night shifts) and who suffered from sleep deprivation (sleeping few hours and feeling unrefreshed after sleep), were at higher risk of stress. The findings of this study replicate findings from previous studies that used various stress measurement tools to identify the parameters associated with higher stress in residents, such as prolonged working hours, high patient load, critical patients assigned, night duty, poor sleep duration, and quality, poor work environment, and process failure [13,16]. The importance of prolonged working hours in causing fatigue and sleep deprivation, which consequently caused stress, led to the legal restriction of residents’ weekly working hours in the United States in 2003 [23]. This restriction probably had a positive impact on the well-being of the residents. Nonetheless, another study has shown that prolonged working hours may be responsible for both stress and decreased job satisfaction among residents. Unfortunately, most of the residents never received efficient or professional stress management.

The stressors associated with stress in this study covered the 3 groups of stressors described earlier: institutional, professional, and personal stressors [10]. The respondents’ stress was associated with dissatisfaction with colleagues and dissatisfaction with training program, income, and frequent thoughts of quitting the medical profession. This dissatisfaction might cause the stress or vice versa, which indicates a need for stress management programs during residency.

In conclusion, the degree of work-related stress among residents in Jordanian hospitals is considerably moderate to high. The most important significant risk factors identified by multiregression analysis were the following: facing work-related stressors, academic-related stressors, family-related stressors, and uncomfortable work environment. Establishment of professional counseling for the residents is highly recommended to deal with their issues in a timely manner that would support their needs. This could lead to an enhancement in the working environment.

## Abbreviations

PSS: perceived stress scale
